# High cryptic species diversity is revealed by genome-wide polymorphisms in a wild relative of banana, *Musa itinerans,* and implications for its conservation in subtropical China

**DOI:** 10.1186/s12870-018-1410-6

**Published:** 2018-09-14

**Authors:** Wei Wu, Wei-Lun Ng, Jun-Xin Yang, Wei-Ming Li, Xue-Jun Ge

**Affiliations:** 1 State Key Laboratory of Biocontrol, Sun Yat-sen University, Guangzhou, 510275 China; 2 Center for Environmental Remediation, Institute of Geographic Sciences and Natural Resources Research, Chinese Academy of Sciences, Beijing, 100101 China; 3 Key Laboratory of Tropical Fruit Biology, Ministry of Agriculture, South Subtropical Crops Research Institute, Chinese Academy of Tropical Agricultural Sciences, Zhanjiang, 524091 China; 4 Key Laboratory of Plant Resources Conservation and Sustainable Utilization, South China Botanical Garden, the Chinese Academy of Sciences, Guangzhou, 510650 China

**Keywords:** Crop wild relatives (CWRs), Genome resequencing, *Musa*, Species delimitation

## Abstract

**Background:**

Species delimitation is a challenging but essential task in conservation biology. Morphologically similar species are sometimes difficult to recognize even after examination by experienced taxonomists. With the advent of molecular approaches in species delimitation, this hidden diversity has received much recent attention. In addition to DNA barcoding approaches, analytical tools based on the multi-species coalescence model (MSC) have been developed for species delimitation. *Musa itinerans* is widely distributed in subtropical Asia, and at least six varieties have been documented. However, the number of evolutionarily distinct lineages remains unknown.

**Results:**

Using genome resequencing data of five populations (making up four varieties), we examined genome-wide variation and found four varieties that were evolutionary significant units. A Bayesian Phylogenetics and Phylogeography (BP&P) analysis using 123 single copy nuclear genes support three speciation events of *M. itinerans* varieties with robust posterior speciation probabilities; However, a Bayes factor delimitation of species with genomic data (BFD*) analysis using 1201 unlinked single nucleotide polymorphisms gave decisive support for a five-lineage model. When reconciling divergence time estimates with a speciation time scale, a modified three-lineage model was consistent with that of BP&P, in which the speciation time of two varieties (*M. itinerans* var. *itinerans* and *M. itinerans* var. *lechangensis*) were dated to 26.2 kya and 10.7 kya, respectively. In contrast, other two varieties (*M. itinerans* var. *chinensis* and *M. itinerans* var. *guangdongensis*) diverged only 3.8 kya in the Anthropocene; this may be a consequence of genetic drift rather than a speciation event.

**Conclusion:**

Our results showed that the *M. itinerans* species complex harbours high cryptic species diversity. We recommend that *M. itinerans* var. *itinerans* and *M. itinerans* var. *lechangensis* be elevated to subspecies status, and the extremely rare latter subspecies be given priority for conservation. We also recommend that the very recently diverged *M. itinerans* var. *chinensis* and *M. itinerans* var. *guangdongensis* should be merged under the subspecies *M. itinerans* var. *chinensis.* Finally, we speculate that species delimitation of recently diverged lineages may be more effective using genome-wide bi-allelic SNP markers with BFD* than by using unlinked loci and BP&P.

**Electronic supplementary material:**

The online version of this article (10.1186/s12870-018-1410-6) contains supplementary material, which is available to authorized users.

## Background

Species are the basic units of biodiversity, and precise species delimitation is essential for unbiased biodiversity estimation [1]. While there is no perfect species concept, it is generally agreed that species should be delimited as evolutionarily distinct lineages, usually evident by significant morphological, genetic, or niche differentiation [2, 3]. Today, the vast majority of species are still recognized based on morphological differences alone, and many genetically distinct but morphologically similar species remain undetected. These cryptic species make up a significant proportion of hidden diversity: one estimate suggest that about 60% of 408 newly described mammal species between 1993 and 2009 are cryptic species [4]. The presence of cryptic species is related to both the intrinsic properties of organisms and with extrinsic environmental conditions [1, 3], thus making the discovery of cryptic species extremely challenging.

With the advent of cheap and rapid DNA sequencing, species delimitation using molecular data has become active and fruitful [5]. Until recently, cryptic species were identified using molecular data mainly based on estimating reciprocal monophyly or genetic distances. Comparing DNA barcodes (such as the *mat*K and *rbc*L genes in plants and the *COI* gene in animals) using the Automatic Barcode Gap Discovery (ABGD) program is an efficient and popular means of cryptic species identification [6–8]. This method, which relies on a ‘barcode gap’ within and between taxa, however, becomes impossible if a prior reference library is unavailable or if the barcode gap is unclear. In addition to these simple pairwise distance threshold methods, multi-species coalescent (MSC)-based methods tracks the genealogical histories of samples back to common ancestors and identify possible evolutionarily independent lineages using both Bayesian and maximum likelihood (ML) methods [9]. Accommodating the uncertainties in gene trees, the Bayesian Phylogenetics and Phylogeography program (BPP or BP&P) jointly estimates the posterior probability distributions of different species delimitation models and relevant parameters, including coalescent times and population sizes [10–13]. This program has been constantly updated and has been widely used in species delimitation studies of various taxonomic groups, including plants [14], birds [15], and insects [16]. However, it ignores ongoing gene flows between populations, and is prone to lump distinct species into one species [17]. Divergence with gene flow is very common in incipient speciation [18, 19], and complicates species delimitation. Although a phylogeographic model test program that considers gene flow in a flexible model space has been developed (i.e. Phylogeographic Inference using Approximate Likelihoods, PHRAPL) [20, 21], the authors themselves have pointed out that PHRAPL may not be as powerful as BP&P in delimiting species with deep divergence times or weak migration rates.

So far, species delimitation studies using coalescent theory with Bayesian or likelihood methods have generally been limited to using datasets consisting of dozens of loci [14]. This amount of data is insufficient for the detection of some shallowly diverged lineages [22]. With the ever-decreasing cost of high-throughput sequencing and improved computational power, genomic data has become more accessible for species delimitation studies. Restriction site associated DNA sequencing, (RADseq) methods [23], for example, provide ample random single nucleotide polymorphisms (SNPs) and have been used to generate genome-wide datasets for species delimitation in non-model organisms [24, 25]. To circumvent the computational challenges inherent in such genomic-enabled species delimitation approaches, species trees are often inferred directly from biallelic markers (e.g., SNP or AFLP data) without genes trees [26]. For instance, the program Bayes Factor Delimitation (*with genomic data) (BFD*) estimates the probability of allele frequency change across ancestor/descendent nodes, and obtains a posterior distribution for the species tree, species divergence times, and effective population sizes simultaneously [27].

*Musa itinerans* Cheesman (Musaceae) is a giant perennial monocotyledonous herb named after its long rhizome. It is also a wild relative of the edible banana progenitors, *M. acuminata* and *M. balbisiana*, and is distributed throught Southeast Asia and subtropical China. *Musa itinerans* is morphologically highly variable and as many as six varieties have been proposed, some of which are varieties that were formerly considered to be subspecific [28]. The distribution of these varieties spans over a large area within the monsoonal climate in subtropical China. Since it is tolerant to both frost and drought [28], *M. itinerans* is one of the species in the genus *Musa* that is most resistant to *Fusarium* wilt disease, the Tropical Race 4′ (*Fusarium oxysporum* f. sp. *cubense* race 4, Foc-TR4) [29]. Wild *M. acuminata* species are well-known for having diverse subspecies, some of which are associated with early banana cultivars [30]; in contrast, many banana cultivars show a lack of genetic diversity mainly due to clonal propagation over many generations, that has made them vulnerable to a variety of diseases. Hence, because of its close relationship to banana and its tolerance to a diverse range of biotic and abiotic stresses, *M. itinerans* holds great promise for the improvement of important agronomic traits in banana breeding. To aid in the conservation of genetic resources of *M. itinerans*, it’s important to ascertain the cryptic diversity present in this highly variable species complex. Recognizing the presence of cryptic species also provides opportunities to understand the evolutionary and ecological processes driving the diversification of the genus *Musa* [1].

The *M. itinerans* species complex is composed of six morphologically differentiated varieties, and a draft genome for *Musa itinerans* var. *itinerans* has been previously reported [31]*.* In this study, we sampled four varieties across different latitudes in South China and obtained genome-wide SNP data by genome resequencing. Using this data, we try to answer the following specific questions: (1) Are the varieties represent independent evolutionary lineages or only products of phenotypic plasticity? (2) What’s the real taxonomic status of these lineages under the MSC species delimitation framework?

## Results

### Genome-wide polymorphisms among different morphological species

Resequencing of 24 *M. itinerans* individuals from five populations generated a total of 2.75 billion filtered pair-end reads (249.7 Gb of filtered bases), and these short reads were mapped against the reference genome of *M. itinerans* (http://banana-genome-hub.southgreen.fr/organism/Musa/Itinerans) with a mean unique mapping depth of 15.5, and coverage of 86.9%, (Additional file 1: Table S1). After SNP calling, 9,402,402 SNPs of the 336,835,601 effectively mapped sites passed filtering our criteria.

**Fig. 1:**
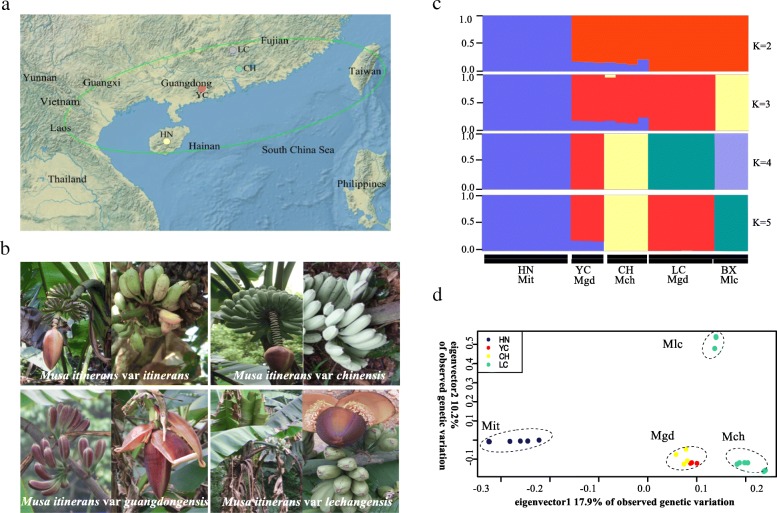
Sampling information and population structure. (**a**) Sampling locations of *Musa itinerans* varieties used in this study: LC, Lecang, Guangdong; CH, Conghua, Guangdong; YC, Guangdong; HN, Hainan; The distributions of *M. itinerans* are outlined by green circles; (**b**) Morphological characters of *M. itinerans* varieties; (**c**) Plots of membership clusters for varieties of *M. itinerans* using genome wide single nucleotide polymorphism data implemented with fastSTRUCTURE. (**d**) Principal components analysis of four geographic populations of *M. itinerans* based on the same genome-wide dataset

**Fig. 2:**
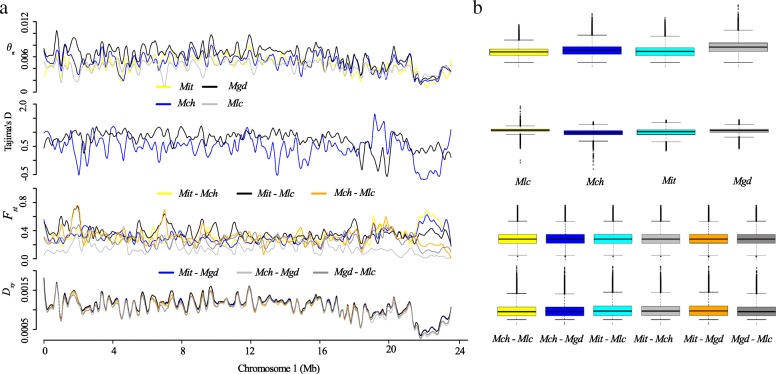
Genome diversity among the four varieties of *Musa itinerans.* (**a**) The distributions of average pairwise nucleotide diversity θ_π_, Tajima’s D, Wright’s fixation index *F*_*ST*_ and absolute genetic divergence *D*_*xy*_ across chromosome 1 with an overlapped window size of 20 kb and a step size of 2 kb for four varieties of *Musa itinerans*; (**b**) Boxplots shown for the overall θ_π_, Tajima’s D, Wright’s fixation index *F*_*ST*_ and absolute genetic divergence *D*_*xy*_ for four varieties of *M. itinerans*

Using a data of 7,940,468 SNP sites without missing genotype, a variational Bayesian inference method implemented in the program fastSTRUCTURE [32] with logistic priors was used to estimate the optimal ancestral components of individuals from different geographic populations. When *K* = 2, samples of *M*. *itinerans* var. *itinerans* (abbreviated as ‘*Mit’* hereafter) from the HN population were separated from the remaining continental populations (i.e. YC, LC: *M. itinerans* var. *guangdongensis*, ‘*Mgd*’; CH: *M. itinerans* var. *chinensis,* ‘*Mch*’; and BX: *M. itinerans* var. *lechangensis*, ‘*Mlc*’). When *K* was increased to 3, the variety *Mlc* (BX) clustered out as a distinct lineage. At *K* = 4, four varieties (*Mit*, *Mlc*, *Mch*, and *Mgd*) were distinguishable from each other. At *K* = 5, the two allopatric populations of *Mgd* were further divided (Fig. 1c). Principal component analysis (PCA) showed strong population structuring (Tracy-Widom statistics: *P* < 1 × 10–^12^), with *Mit* and other mainland varieties separated by the first eigenvector, followed by *Mlc* being clustered out from other varieties by the second eigenvector (Fig. 1d). According to the optimal clusters given by fastSTRUCTURE, we used *K* = 4 and estimated genome-wide diversities using the two common statistics, *θ*_*π*_ [33] and Tajima’s *D* [34]. We found that the two marginally distributed varieties *Mit* (mean *θ*_*π*_ = 4.6 × 10^− 3^) and *Mlc* (mean *θ*_*π*_ = 4.3 × 10^− 3^) harbored significantly fewer variation than other varieties (mean *θ*_*π*_ = 5.1 × 10^− 3^, and 6.4 × 10^− 3^ for *Mch* and *Mgd* respectively, *P* < 2.2 × 10^− 16^, Mann Whitney U-test; Fig. 2, Additional file 1: Figure S1 and S2). The overall positive genome-wide Tajima’s *D* values for all varieties (the median value of *D*_*mit*_ = 0.69, *D*_*mgd*_ = 0.87, *D*_*mch*_ = 0.55, *D*_*mlc*_ = 0.94, Fig. 2 and Additional file 1:Figure S1 and S2) indicated that *M. itinerans* probably experienced population contractions in the past.

The values of genome-wide population differentiation *F*_*ST*_ [35] among the four varieties ranged between 0.14 and 0.41, with *Mit* showing higher differentiation with any of the other varieties, which was consistent with the genome-wide absolute genetic divergence *D*_*xy*_ [36] (mean D_xy_ = 0.0008–0.0009; Table 1). *D*_*xy*_ showed lower levels of variation among different variety pairs (Fig. 2, Additional file 1: Figure S1 and S2), as it is insensitive to current levels of polymorphism within species and reflects the net divergence since their common ancestor [37].

Analyses of the *D*-statistics calculated from the genome-wide dataset revealed that historical gene flow occurred between different varieties (Table 2). Low values of absolute *D*-statistic were found in comparisons of *Mlc-Mch* (*D* = 0.04) and *Mlc-Mit* (*D* = − 0.08) respectively, which indicating infrequent gene flow that may have facilitated the formation of *Mlc* as a distinct lineage. In contrast, higher absolute *D* values (− 0.15, − 0.19) suggested that significant gene flow has occurred between the *Mit* and *Mch* varieties after divergence. Considering their current allopatric distribution, this historical gene flow may have occurred before the formation of the Qiongzhou Strait (about 10.3 kya), which isolated Hainan island from the continent [31].

### Bayesian species delimitation

**Fig. 3:**
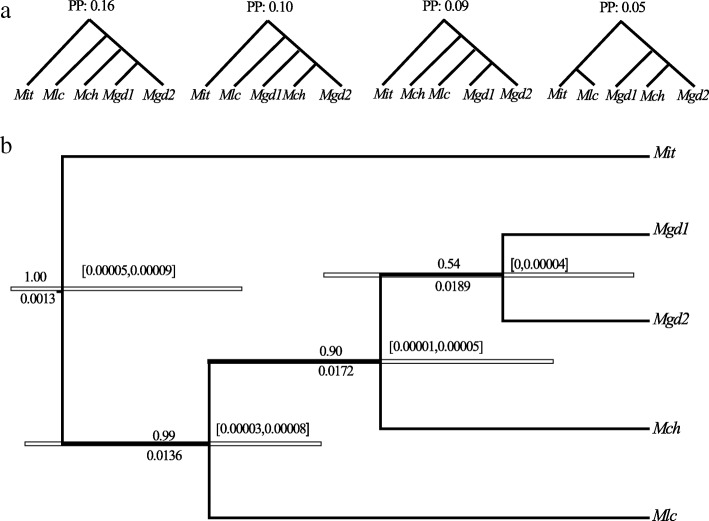
Species delimitation for *Musa itinerans* varieties using Bayesian phylogenetics and phylogeography (BP&P) based on 123 single copy nuclear loci. (**a**) Specie tree estimation: the top four species trees and their posterior probabilities with a total probability of 0.5, and population sizes with Gamma priors θ ~ G (2, 1000) for all populations and divergence times with Gamma priors τ ~ G (2, 2000) for the root age. The abbreviations for different varieties or geographical populations are as follow: *Mit*: *Musa itinerans* var. *itinerans*; *Mlc*: *Musa itinerans* var. *lechangensis*; *Mch*: *Musa itinerans* var. *chinensis*; *Mgd*1: *Musa itinerans* var. *guangdongensis* (population Yangchun, Guangdong); *Mgd*2: *Musa itinerans* var. *guangdongensis* (population Lechang, Guangdong); (**b**) species delimitation on guide tree: above and below the branches are the posterior speciation probabilities, population sizes are shown on every node, the 95% highest posterior density (HPD) for divergence time are in the brackets and are highlighted using horizontal grey bars. Two geographical populations of *M. itinerans* var. *guangdongensis* were lumped with weak posterior probability, the splits of other varieties were supported with high posterior probabilities

We performed the BP&P analysis at *K* = 5, that is composed of *Mit*, *Mch*, *Mlc*, and two allopatric populations of *Mgd*. Based on this pattern of clustering, the Bayesian species tree estimation yielded 97 distinct species trees, of which the top 40 species trees constituting a 95% credibility set of tree topologies. The majority-rule consensus tree is almost star-shaped, suggesting that these varieties diverged very recently. We used the tree with maximum posterior probability of 0.16 (Fig. 3a), in which two geographical populations of *Mgd* were monophyletic and most closely related to the variety *Mch,* followed by *Mlc.* Using this five-lineage phylogeny as a guide tree, the posterior probabilities of different models and the posterior distribution of the parameters *τ*_s_ and *θ*_*s*_ for each model were calculated using the rjMCMC algorithm. The five-lineage and four-lineage models yielded posterior probabilities of 0.54, and 0.36, respectively. Thus, the maximum a posterior probability (MAP) model uses a five-lineage model as the guide tree (Fig. 3b). Nonetheless, the posterior speciation probability of the node for the two geographic populations of *Mgd* (YC, LC) was 0.54, far below the conservative threshold of 0.95, showing weak evidence for a split of this variety. The posterior speciation probability of the node with *Mch* and *Mgd* was 0.90, somewhat below the threshold of 0.95, indicating that it is possible to plausibly lump the two varieties (*Mch* and *Mgd*) together. Considering the high number of loci (123 genes) used in our coalescence analysis, this is likely an indication of recent divergence of these varieties rather than insufficient variation detected in our study. Two other varieties *Mit* and *Mlc,* seemed to be well resolved lineages (i.e. the posterior speciation probability was 1.00 for *Mit*, and 0.99 for *Mlc*). Overall, high posterior speciation probabilities supported the three-species delimitation scenario by two speciation events among the four varieties. Using the same gamma prior *θ* ~ G (2, 1000) for population sizes and divergence time with *τ*_*0*_ ~ G (1, 10,000) at the root to estimate the parameters in the multi-species coalescent model for the MAP tree, the posterior mean of the population size parameter *θ*_*s*_ ranged from 0.0013 to 0.0180, and population size-scaled divergence time at the root was 7.0 × 10^− 5^ (95% confidence interval: 5.0 × 10^− 5^ ~ 9.0 × 10^− 5^), and other nodes ranged between 2.0 × 10^− 5^ and 9.0 × 10^− 5^.

**Fig. 4:**
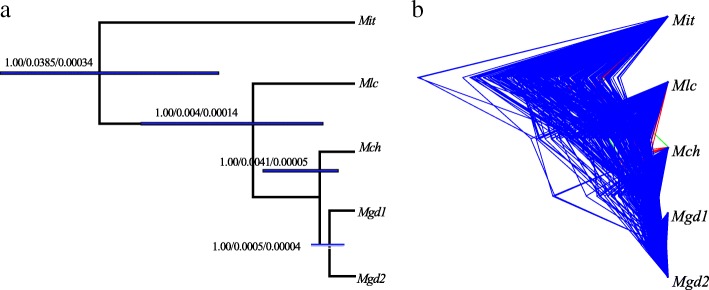
Bayesian factor species delimitation for *Musa itinerans* varieties using 1201 unlinked loci. (**a**) the five taxa species tree determined by the most marginal likelihood estimates of different species models. Bayesian posterior probability, ancestral population size, divergence time are separated by two slashes; the 95% highest posterior density (HPD) for divergence times are highlighted using horizontal grey bars; (**b**) DensiTree shown for all trees of the Markov chain Monte Carlo method with a burn-in of 5000 trees, and higher levels of uncertainty are represented by lower densities

Bayesian factor delimitation methods also gave decisive support for the five-lineage model with an BF value of 81.4, well above the threshold of 6, supports the hypothesis that the two allopatric geographic populations of *Mgd* show evolutionary divergence and are therefore evolutionarily distinct lineages (Table 3)*.* Bayesian analyses of the 1201 unlinked SNPs yielded a well-resolved species tree for all currently recognized varieties (Fig. 4, all nodes have a posterior probability of 1.0). The divergence age at the root of the species tree was estimated to be 0.00034 (95% CI: 0.00018 ~ 0.00048). Using a generation time of one year and base substitution rate of 1.30 × 10^− 8^ substitution rate per site per year [38], we estimate that this divergence occurred approximately 26.2 kya (13.8 ~ 36.9 kya), i.e. during the Late Pleniglacial period, when drastic climatic fluctuations and associated habitat alterations profoundly altered the speciation rates in marginal tropical areas. In Hainan, the final formation of the Qiongzhou Strait during the mid-Holocene (7.0 ~ 10.5 kya) [39] may have further facilitated the divergence between *Mit* and its continental counterparts. The speciation time of *Mlc* also date to the Holocene (*Mlc*: 10.7 kya, 95% CI: 3.1 ~ 22.3 kya), and this variety has the most northerly distribution in South China. Moreover, it is the most frost-tolerant variety and may show some degree of ecological speciation. The divergence time of *Mch* and *Mgd* was dated to 3.8 kya (95% CI: 1.5 ~ 9.2 kya). Finally, the divergence of the two allopatric populations *Mgd* was dated to 3.1kya (95% CI: 0.1 ~ 4.6 kya), and we speculate that this very recent divergence should is more likely a consequence of genetic drift in the Anthropocene rather than a result of speciation. By lumping the two geographical populations of *Mgd* together, and merging the two varieties of *Mgd* and *Mch*, both the BP&P and BFD* approaches agreed on aa consensus species delimitation scenario for the varieties of *M. itinerans*.

## Discussion

### Four *M. itinerans* varieties are evolutionarily significant units

In this study, genome-wide SNP data was used to reveal the cryptic diversity within the *M. itinerans* species complex*.* All four sampled *M. itinerans* varieties were shown to be genetically divergent and to represent evolutionarily distinct lineages. This species is one of the most widely distributed wild relatives of banana in the subtropical region, moreover, it harbors great intraspecific genetic diversity and has potential use for the improvement of disease resistance in banana [29]. So far, 7–8 varieties of *M. itinerans* with distinct morphological characters have been documented by taxonomists [28, 40–42]. However, it is unclear how many of these are genetically distinct lineages. ‘Variety’ is a taxonomic rank below ‘subspecies’ [43] and is commonly used when range-wide populations of a species exhibit recognizable morphological differences, often in response to fluctuation environments. On the other hand, evolutionarily significant units (ESUs) are populations that do not undergo frequent genetic exchange, and hence should display reciprocal monophyly and significant divergence of allele frequencies at nuclear loci [44]. In conservation biology, the recognition of ESUs is relevant to defining conservation priorities and strategies.

Genetic component inferences based on a variational Bayesian framework and on a principal component analysis provided compelling evidence that the four varieties found in South China are distinct evolutionary lineages. The four varieties showed significant genetic differentiation (*F*_*ST*_: 0.14 ~ 0.40), which was further validated by the BP&P and BFD* species delimitation analyses. The BP&P analysis supported the three-lineage model (*Mit*, *Mlc*, and *Mch + Mgd*1 + *Mgd*2), while the BFD* analysis supported the five-lineage model (*Mit*, *Mlc*, *Mch*, *Mgd*1, and *Mgd*2). However, the BFD* analysis also provided estimated divergence times between *Mch* and *Mgd*, as well as between the two allopatric *Mgd* populations, that are very recent - 3.8 and 3.1 kyr respectively. These divergence times are beyond a reasonable time scale for speciation. Considering both observations, we propose that two varieties, *Mit* in Hainan and *Mlc* in northern Guangdong, be elevated in rank to subspecies. According to Häkkinen’s descriptions [28], *Mit* was easily distinguished from the other varieties by its long creeping rhizome, which often extends as far as 2 m away from the mother plant; In addition to its narrow distribution, *Mlc* also presents with obtuse female buds and small purple blotches on its pseudostems. Hence, it seems that rhizomes and suckers can be used as diagnostic traits for the two subspecies. The other two varieties, *Mch* and *Mgd,* should be merged and named as the subspecies *M. itinerans* subsp. *chinensis* according to their publication date. *Mch* was found to significantly differ from the other varieties by its denser suckers and the presence of large red-brown blotches on its pseudostems. *Mgd* is recognized by its green to purple fruit peel when ripe [28]. These traits may be due only to morphological plasticity of a single subspecies, *M. itinerans* ssp*. chinensis.* The subspecies *Mlc,* which has a sporadic distribution, was proposed to be categorized as a ‘Vulnerable’ (VU) species for conservation. This subspecies has been observed to be capable of withstanding frost damage [28], highlighting the importance of its conservation as an important genetic resource. *Mlc* diverged from the ancestor of *Mgd* and *Mch* about 10.7 kya (95% CI: 3.1 ~ 22.3). This divergence was followed by population contractions (as indicated by a positive Tajima’s *D*), indicating that the *Mlc* may be a remnant from a larger population in the past.

### Unlinked biallelic SNPs or multiple unlinked loci in shallow species delimitation?

We used two analytical tools based on the MSC model to test possible species delimitation schemes for the varieties of *M. itinerans*. The BP&P method favoured a three-lineage model, whereas the BFD* approach initially supported a five-lineage model; the main difference was the BFD* approach identified the very recently diverged *Mgd* and *Mch* varieties as distinct lineages. However, when reconciling the divergence time estimates with plausible time scales of speciation, the BFD* approach also supported to a three-lineage model. The relative power of the different methods involves many factors, including the number of unlinked loci or SNPs, the prior assignments of individuals, and the prior settings of parameters space. In this study, the same cluster assignments were used in both approaches, and the parameters were optimized according to the same polymorphism estimates, leaving the numbers of markers used in the two datasets as the major variable that differed between the two approaches. The BP&P method is known to be more conservative and may be prone to species lumping when divergence is recent [15], as observed in the case here with the *Mch* and *Mgd* varieties. The BP&P approach has been shown to be capable of validating species boundaries between well-differentiated species using even one locus [45], and this is of great advantage in revealing cryptic species diversity in poorly resolved taxa. For recent speciation events, hundreds or thousands might improve the power of BP&P where computational resources are available, but such cost is not readily achieved in non-model organisms [22]. In our study, the performance of the BFD* approach using 1201 unlinked SNPs was comparable to using 123 unlinked loci. Since the cost of obtaining this many unlinked SNPs using RADseq was lower than collecting 123 unlinked loci in a non-model organism, it may be most efficient to use the bi-allelic markers with the BFD* method for species delimitation in recently diverged lineages. With ever-decreasing costs, the genotyping of thousands of loci using RADseq may someday become a routine practice and would therefore facilitate the discovery of cryptic species present after recent speciation events. Such an approach is also of paramount importance for the conservation of understudied CWRs.

### Population structure or ESUs?

Population genetic structures persist in all taxa along their geographic and/or environmental barriers. One puzzle in species delimitation is how to distinguish population structure from real speciation events of divergent lineages [46]. To avoid misidentification structured populations as separate species, external information (i.e. a priori hypothesis or knowledge) is essential to interpret the genetic data and provide a basis on which to judge whether differences corresponding to different species rather than different populations of a single species. In this study, although the *Mlc* variety of the BX population and the *Mgd* variety of the LC population are sympatric, their divergence was found to be much higher than the genetic differentiation between the two allopatric *Mgd* populations. This result - the opposite of what might be expected from the isolation by distance model - indicated that the genetic divergence observed in our study was between ESUs rather than merely between populations separated by distance. In addition to obvious morphological differences, the two varieties differed remarkably in habitat preference (i.e. warm and humid vs. cold and dry microhabitats) [26].

## Conclusion

Overall, classification of plants within the genus *Musa* is difficult even for skilled taxonomists, and new taxa have been reported mainly based on morphological characters [40–42, 47–49]. This study demonstrated the feasibility of using high-throughput sequencing data, in association with biological information such as morphological characters, biogeographic history, and ecological differentiation for the delimitation of ESUs within the genus *Musa*. The currently recognized *M. itinerans* var. *itinerans* and *M. itinerans* var. *lechangensis* should be elevated in rank as subspecies for better management and conservation of these taxa as CWRs genetic resources. Species delimitation using molecular approaches can free the taxonomists from being entangled with subtle character differences that may mask differences in real. However, species delimitation using molecular approaches should be cautious and consider biologically meaningful data, such as morphological characters, biogeographic history, and ecological differentiation. As the case in our BFD* analysis, the estimated divergence time for the *Mch* and *Mgd* varieties was not a reasonable time scale for real speciation to have taken place.

## Methods

### Sample collection, resequencing, and filtering of raw reads

According to the assigned variety scheme present at the outset of our study [28], we sampled four varieties of *M. itinerans* from five populations across South China (Fig. 1a and b, Table 4). Fresh leaves were harvested in the field and dried using silica-gel. Total genomic DNA was extracted using the standard CTAB extraction method. A library with 500 bp insertion size for each individual plant was prepared using Paired-End Sample Prep Kits (Illumina, UK) according to the manufacturer’s recommendations, libraries were then sequenced on the HiSeq2000 platform. Using NGSQCToolkit version 2.3.3 [50], raw reads were discarded with excessive (> 10%) ‘N’s or over 40% of the bases with PHRED quality score below 7 were discarded. Raw data has been deposited in the NCBI Sequence Read Archive under BioProject ID 312694 with accession number SRR6382516~SRR6382539.

**Table 4:** Sample location information for specimens used in this study

Taxon (abbreviation)	Location	Coordinate (Altitude: a.s.l)	Sample size	Voucher specimens
*Musa itinerans* var. *itinerans* (*Mit*)	Jianfengling National Park, Ledong, Hainan	18°43’N, 108°50′E (770 m)	8	Ge201301
*Musa itinerans* var. *guangdongensis* (*Mgd*)	Baiyong Preserve Yangcun, Guangdong	22°23’N, 111°39′E (90 m)	3	Ge201302
*Musa itinerans* var. *chinensis* (*Mch*)	Shimen National Park, Conghua, Guangdong	23°38’N, 113°46′E (200 m)	4	Ge201303
*Musa itinerans* var. *guangdongensis (Mgd)*	Lechang Gorge, Lechang, Guangdong	25°08’N, 113°17′E (150 m)	6	Ge201304
*Musa itinerans* var. *lechangensis (Mlc)*	Beixiang, Lechang, Guangdong	25°20’N, 113°21′E (840 m)	3	Ge201305

**Table 1:** The mean genome-wide genetic differentiation among four varieties of *Musa itinerans* var. *itinerans* (*Mit*)*, M. itinerans* var. *guangdongensis* (*Mgd*), *M. itinerans* var. *chinensis* (*Mch*), and *M. itineran*s var. *lechangensis* (*Mlc*) in South China. The triangular values above show absolute genetic divergence and bellows show the genetic differentiation index *F*_*ST*_

	*Mit*	*Mch*	*Mgd*	*Mlc*
*Mit*		0.0009	0.0009	0.0009
*Mch*	0.29		0.0008	0.0008
*Mgd*	0.26	0.14		0.0008
*Mlc*	0.41	0.31	0.24

**Table 2:** Introgression four population tests of varieties of *Musa itinerans*

JK-D^a^	V (JK-D)^b^	Z-score^c^	*P*-value	nABBA	nBABA	nBlocks	^d^Four populations
−0.19	0.000069	−31.81	0	116,130.9	171,401.0	97	((*Mch*, *Mgd*), *Mit*), O)
−0.08	0.000041	−12.28	0	140,626.9	164,749.7	97	((*Mch*, *Mgd*), *Mlc*), O)
−0.15	0.000059	−19.63	0	119,522.4	161,999.8	97	((*Mch*, *Mlc*),*Mit*), O)
0.04	0.000054	5.33	0	149,391.0	138,090.1	97	((*Mgd, Mlc*), *Mit*), O)

^a^JK-D denotes the mean value of Jackknife bootstrapping;

^b^V(JK-D) denotes the variance of JK-D;

^c^Z scores > 3 are considered significant evidence of a nonzero D-statistic value, consistent with the presence of admixture; given phylogeny (((P1, P2), P3), O)), positive D-statistic values are indicative of gene flow between P2 and P3; negative values are indicative of gene flow between P1 and P3

^d^*Mch*: *M. itinerans* var. *chinensis, Mgd*: *M. itinerans* var. *guangdongensis, Mlc*: *M. itinerans var. lechangensis, Mit*: *M. itinerans* var. *itinerans,* O: outgroup, *M. basjoo*

**Table 3:** BFD* species delimitations for the varieties of *Musa itinerans*

Model	Species Number	MLE	Rank	BF
RunD, *Mch, Mlc, Mgd, Mit,* current model	4	− 3667.1	4	–
RunB, *Mch, Mlc, Mgd* lumped	2	− 3649.5	2	−35.2
RunC, *Mch, Mgd* lumped	3	− 3722.4	3	54.9
RunE, two populations of *Mgd* split	5	− 3626.4	1	−81.4

Bayes factor (BF) calculations were made against the current taxonomy model (RunD), positive BF values indicate support for the current taxonomy model, and negative BF values indicate support for the alternative model

### Read alignment, variant calling, and variant filtering

For the convenience of calculating or visualizing genome wide diversity statistics, we anchored and oriented the assembled scaffolds of *M. itinerans* against 12 linkage groups of the *M. acuminata* genome based on the collinearity between them, and the remaining scaffold or contigs were treated as one group and ordered by size. Filtered paired-end reads per sample were aligned to the updated reference genome using the BWA-mem version 0.7.12 [51] algorithm with default options. The sam files were converted into bam format, then sorted and indexed using SAMtools version 1.3.1 [52],. Prior to variant calling, MarkDuplicates in Picard (http://broadinstitute.github.io/picard) was used with default options to mark duplicated reads resulting from PCR. After removing reads with mapQ scores below 30 and trimmed lengths less than 30 base pairs (bp), the remaining reads were headed with sample information using Picard-tools, and local realignments around indels were implemented using the IndelRealigner tool packaged in GATK version 3.7.0 [53–55]. Each filtered and aligned file was subjected to variant-calling using the HaplotypeCaller function in GATK, and joint genotyping (by combining all of the above outputs for SNP and indel VCFs) was implemented using GenotypeGVCFs in GATK. Finally, joint variants were filtered using VariantFiltration in GATK using the default settings.

### Population structure and principal component analysis

Using vcftools version 0.1.14 [56], the SNP variants among the 12 linkage groups of three populations were selected with options ‘--maf 0.05’ and were further LD-pruned to minimize the linkage disequilibrium of the sites using the option ‘--indep-pairwise 50 5 0.2’ in PLINK version 1.0.9 [57]. Using this filtered genotype SNP data set, posterior inferences of population structure for these varieties were implemented based on a variational Bayesian framework with the python program fastSTRUCTURE.The Smartpca program packaged in the eigensoft version 6.1.3 [58] was used to perform principal component analysis on the same set of variants using the default parameters.

### Nucleotide diversity and population divergence

A sliding window approach (20-kb overlapped windows in 2-kb steps) was used to quantify genome-wide variation among inferred genetic clusters. We calculated the average pairwise nucleotide diversity, *θ*_*π*_, Tajima’s *D*, Wright’s fixation index *F*_ST_, and the absolute genetic divergence, *D*_*xy*_, using PopGenome version 2.24 [59] or custom Perl scripts. To distinguish between the relative role of lineage sorting and introgressions in the current divergence patterns, an extended *D*-statistics four-population test (ABBABABA2) was conducted using the software package ANGSD-wrapper [60, 61]. Z-scores above 3 was used to reject the null hypothesis of no significant introgression between populations.

### Species delimitation

According to recommendations by Carstens et al. [62], two distinct methodological approaches were used to detect cryptic lineages in varieties of *M. itinerans*, i.e. BP&P and the Bayes factor delimitation method with genomic data (BFD*) [26, 27]. In a previous study, we identified 1201 single copy nuclear genes of *M. itinerans* and other eight related species [29]*,* and different subsets of this data were used in our analyses.

The BP&P3.3a program is a full likelihood-based implementation of the MSC model, and it uses a reversible-jump Markov chain Monte Carlo (rjMCMC) method to evaluate competing delimitation models [10]. It collapses or splits the nodes in the guide species tree according to the nodes posterior probabilities in the guide tree and alternative competing delimitation models [12, 17, 62]. The program consists of four modules: in module A00, it generates the posterior distribution of species divergence times (*τ*_s_) and population sizes (*θs*) under the MSC model with a fixed species phylogeny; in module A01, the species tree is estimated with fixed assignments and species delimitation; in module A10, species delimitation is implemented on a guide tree;and in module A11, the species tree estimation and species delimitation are integrated. According to our genome-wide estimates of nucleotide diversity θ_π_ and absolute divergence time *D*_*xy*_ for the varieties of *M. itinerans* studied here, a small population size with gamma prior *θ* ~ G (2, 1000) for all populations and a shallow divergence time with *τ*_*0*_ ~ G (1, 10,000) at the root of the species tree were assigned, with Dirichlet priors for other divergence time parameters. A total of 2,000,000 iterations (sample interval of 4) with a burn-in of 1000 was implemented for each run. In this study, we followed a multi-steps analysis. Each step was conducted with ten replicates, and convergence was evaluated across replicates. In addition, the convergence of estimation of model parameters was evaluated with the effective sample size (ESS ≥ 200). Because of the computation burden presented by a multi-species coalescence model of thousands of loci, only 123 loci were randomly selected and used for the BP&P analysis.

The BFD* method was performed using SNAPP version 1.3.0 [26, 27]. This program estimates the marginal likelihood of competing models with different numbers of species and individual assignments, and ranks model fit among runs by Bayes factor. The two essential assumptions of this method were that there was no gene flow among lineages and that multiple unlinked loci are used in the coalescence model. Thus, we excluded samples with admixture proportions over 5%; and for the 1201 single copy loci, only sites with maximum calling depth were used to avoid linkage disequilibrium. In our species delimitation model, we lumped or split varieties or geographic populations according to the current classification of varieties and population structure/clustering. The marginal likelihood of each model was estimated via path sampling using 48 steps each with different levels of power-posterior, an alpha of 0.3, and an MCMC chain length of 100,000 with a pre-burn-in of 100,000 [26].

## Additional file

Additional file 1: **Table S1.** Summary of sequencing depth and coverage for each sample accession of *Musa itinerans* used in this study. **Figure S1.** The distributions of overall genome-wide polymorphisms for four varieties of *Musa itinerans.* Data shown for with overlapped window size of 20 kb and step size of 2 kb. **Figure S2.** The distributions of average pairwise nucleotide diversity θ_π_, and Tajima’s *D*, Wright’s Fixation index *F*_*ST*_ and absolute genetic divergence Dxy across chromosome 2 ~ 12. (PDF 4614 kb)

## Abbreviations

BFD *****: Bayes factor delimitation of species with genomic data
BP&P: Bayesian Phylogenetics and Phylogeography program
CI: Confidence Interval
ESUs: Evolutionarily significant units
kya: Thousands of years ago
MAP: Maximum a posterior probability model
MSC: Multi-species coalescence model
PHRAPL: Phylogeographic Inference using Approximate Likelihoods
RADseq: Restriction site associated DNA marker sequencing
SNPs: Single nuclear polymorphisms
