# Supplementary material for: High cryptic species diversity is revealed by genome-wide polymorphisms in a wild relative of banana, Musa itinerans, and implications for its conservation in subtropical China
Source: BMC Plant Biol. 2018 Sep 14;18:194. doi: 10.1186/s12870-018-1410-6 (PMC6137913; doi:10.1186/s12870-018-1410-6)
Supplement: Supplementary file 1 — Table S1. Summary of sequencing depth and coverage for each sample accession of Musa itinerans used in this study. Figure S1. The distributions of overall genome-wide polymorphisms for four varieties of Musa itinerans. Data shown for with overlapped window size of 20 kb and step size of 2 kb. Figure S2. The distributions of average pairwise nucleotide diversity θπ, and Tajima’s D, Wright’s Fixation index FST and absolute genetic divergence Dxy across chromosome 2 ~ 12. (PDF 4614 kb) [file 12870_2018_1410_MOESM1_ESM.pdf]

Table S1 The summary of sequencing depth and coverage for each sample accession of *Musa itinerans* used in this study

			Uniq mapping			Multiple-mapping	
Varieties	Sample accession	Depth (X)	Coverage (%)	Reads	Depth (X)	Coverage (%)	Reads
M. itinerans subsp. *chinensis*	CH1	8.73	85.12	41012748	10.92	90.68	51296411
	CH2	8.95	84.96	42059451	11.22	90.54	52742177
	CH3	8.54	84.97	40133752	10.68	90.5	50195517
	CH4	6.99	84.66	32848354	8.68	90.13	40802440
M. itinerans var. *itinerans*	HN1	20.34	89.82	95553637	24.96	95.18	117223525
	HN2	11.35	88.76	53313501	14.06	94.1	66053039
	HN3	11.08	88.36	52046835	13.74	93.74	64563856
	HN4	13.17	88.83	61861709	16.23	94.2	76240833
	HN5	10.67	87.44	50147590	13.31	92.89	62546088
	HN6	9.18	88.01	43147176	11.37	93.35	53398835
	HN7	8.39	87.56	39400227	10.38	92.85	48746947
	HN9	61.96	94.5	285824400	75.66	99.12	349013348
M. itinerans var. *guangdongensis*	LC1	10.39	85.49	48869340	13.23	91.08	62168683
	LC2	9.87	85.84	46423820	12.46	91.4	58564442
	LC3	8.62	84.83	40514166	10.8	90.42	50776531
	LC4	8.24	85.08	38740464	10.43	90.62	49002962
	LC8	7.29	83.74	34293584	9.14	89.28	42970700
	LC9	8.17	84.04	38409259	10.36	89.62	48723583
	YC1	10.78	86.39	50694500	13.49	91.95	63435851
	YC2	8.75	85.24	41144289	11.01	90.85	51766385
	YC3	54.82	89.61	252505816	68.87	94.82	317147704
M. itinerans var. *lechangensis*	BX1	8.53	86.29	40126714	10.64	91.75	50013421
	BX2	8.72	86.45	40984484	10.93	91.9	51361507
	BX3	48.38	90.35	223335542	59.96	95.48	276712458

(a).

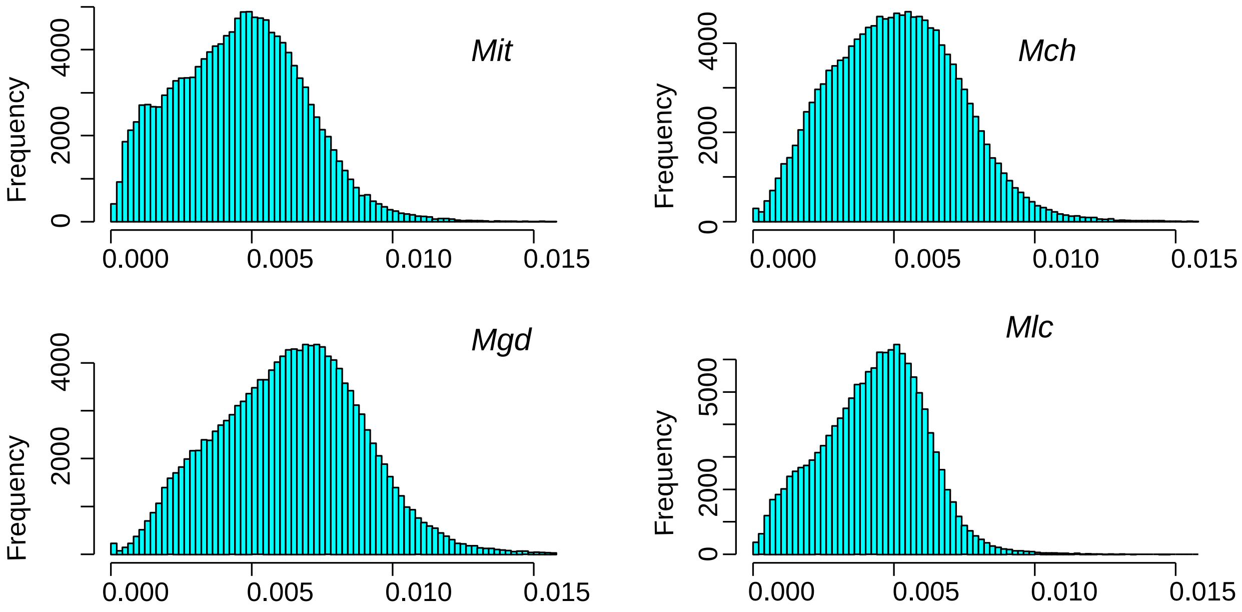

(b)

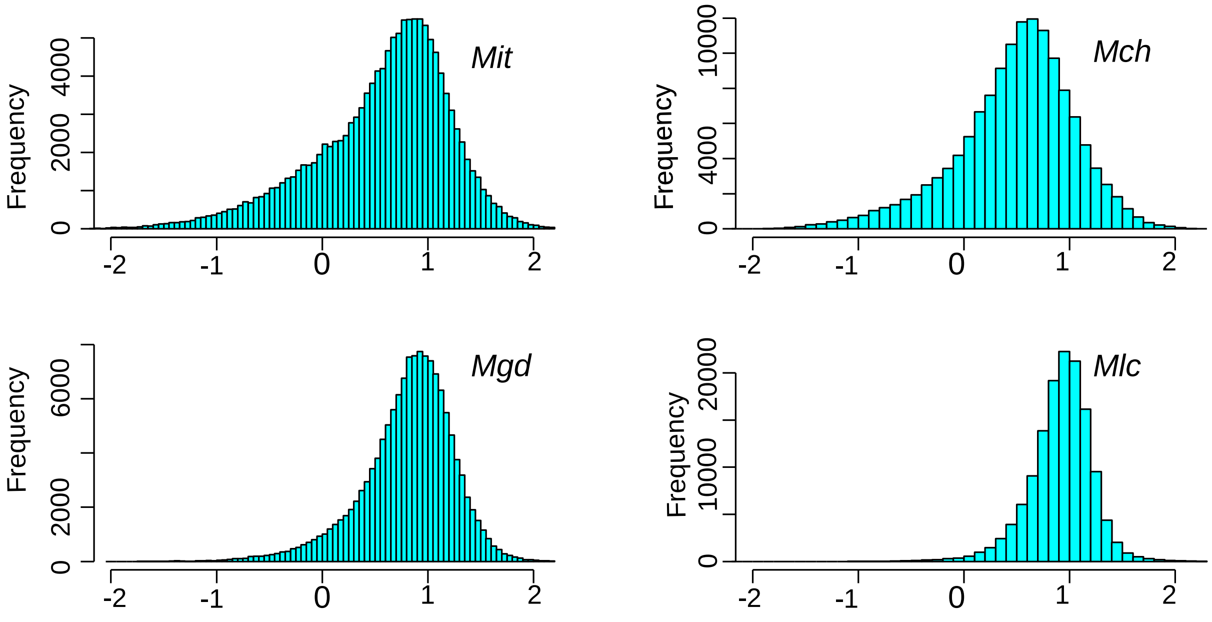

Fig S1. The overall genome-wide polymorphisms distributions with 20kb overlapped window size and 2kb step size among four *Musa itinerans* varieties. (a) pairwise nucleotide diversity θ_{π}, (b) Tajima's D. The the varieties were designated as: *Mit: Musa itinerans* var *itinerans*; *Mch*: *Musa itinerans* var *guangdongensis*; *Mgd*: *Musa itinerans* var *chinensis*; *Mlc*: *Musa itinerans* var *lechangensis*

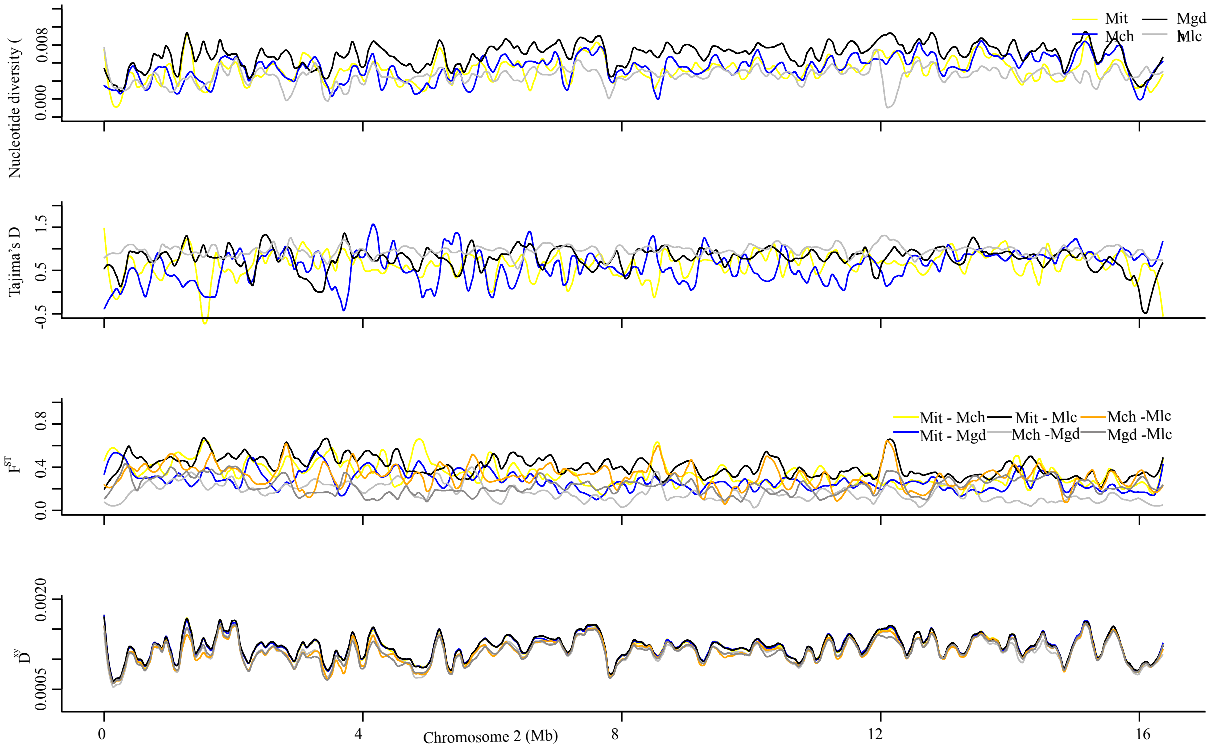
Nucleotide diversity (
0.000
0.008
Mit
Mch
Mgd
Mlc
Tajima's D
-0.5
0.5
1.5
Mit - Mch
Mit - Mlc
Mch -Mlc
Mit - Mgd
Mch -Mgd
Mgd -Mlc
0.0
0.4
0.8
0.0005
0.0020
0
4
8
12
16
Chromosome 2 (Mb)

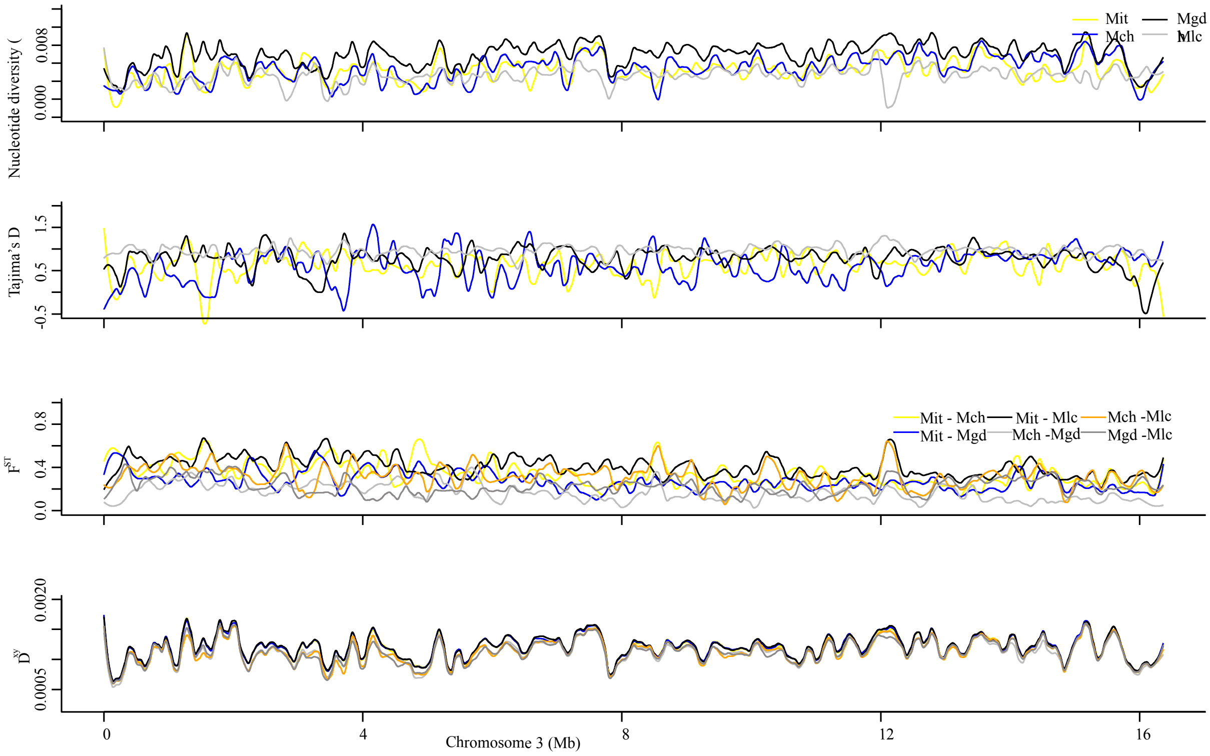
Nucleotide diversity (
0.000
0.008
Mit
Mch
Mgd
Mlc
Tajima's D
-0.5
0.5
1.5
Mit - Mch
Mit - Mlc
Mch -Mlc
Mit - Mgd
Mch -Mgd
Mgd -Mlc
0.0
0.4
0.8
0.0005
0.0020
0
4
8
12
16
Chromosome 3 (Mb)

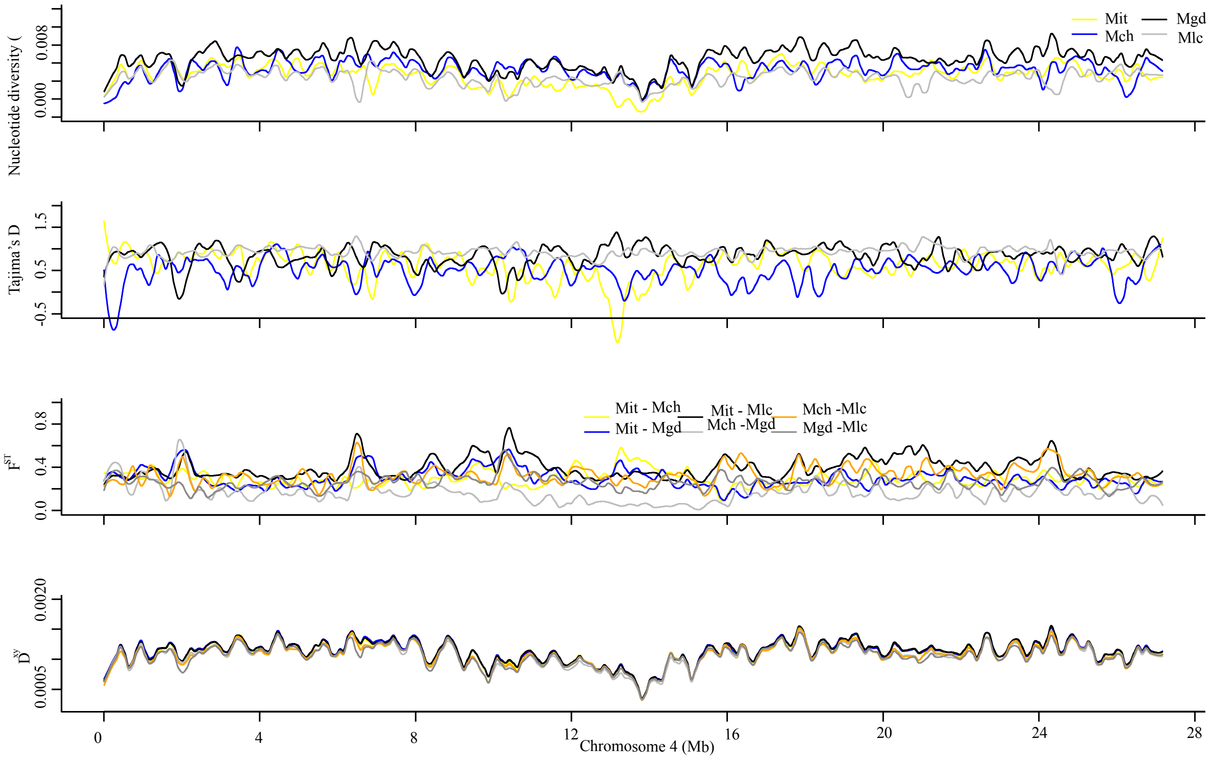
Nucleotide diversity (
0.000
0.008
Mit
Mch
Mgd
Mlc
Tajima's D
-0.5
0.5
1.5
F_{ST}
0.0
0.4
0.8
Mit - Mch
Mit - Mgd
Mit - Mlc
Mch -Mgd
Mch -Mlc
Mgd -Mlc
D_{xy}
0.0005
0.0020
0
4
8
12
16
20
24
28
Chromosome 4 (Mb)

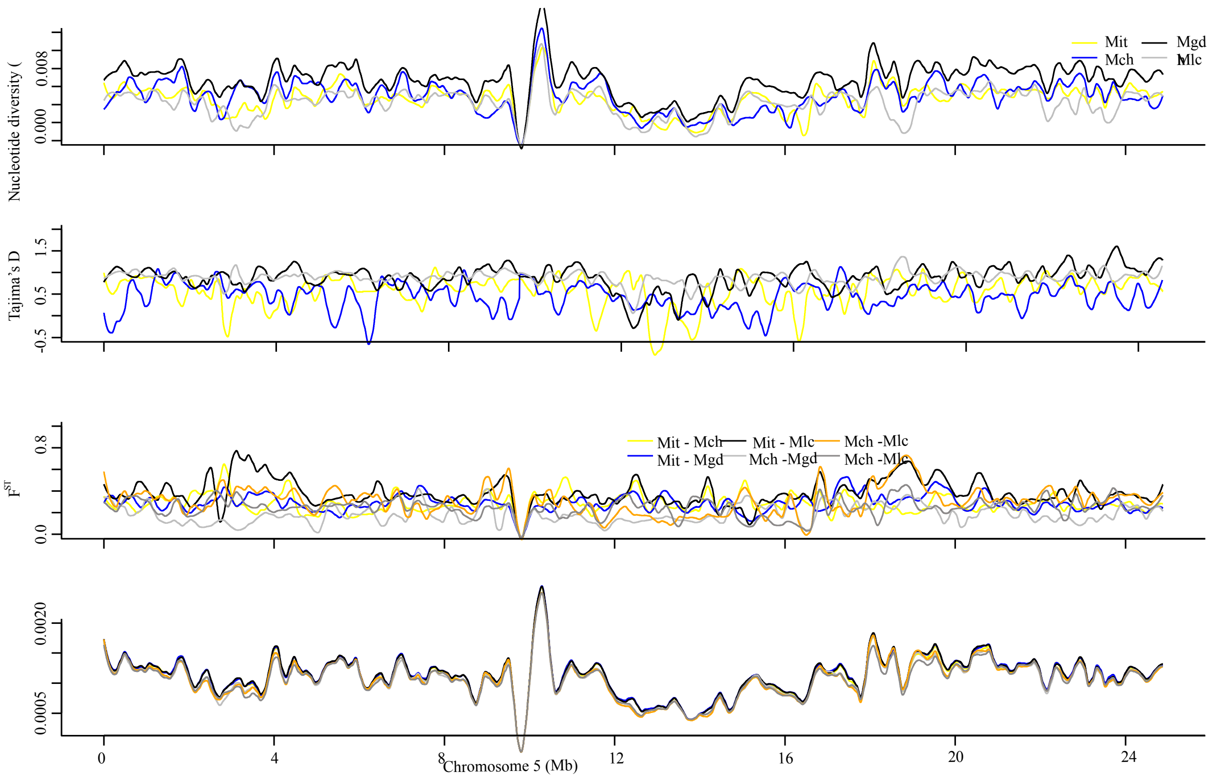
Nucleotide diversity (
0.000
0.008
Mit
Mch
Mgd
Mlc
Tajima's D
-0.5
0.5
1.5
F_{ST}
0.0
0.8
Mit - Mch
Mit - Mgd
Mit - Mlc
Mch -Mgd
Mch -Mlc
Mch -Mlc
0.0005
0.0020
0
4
8
12
16
20
24
Chromosome 5 (Mb)

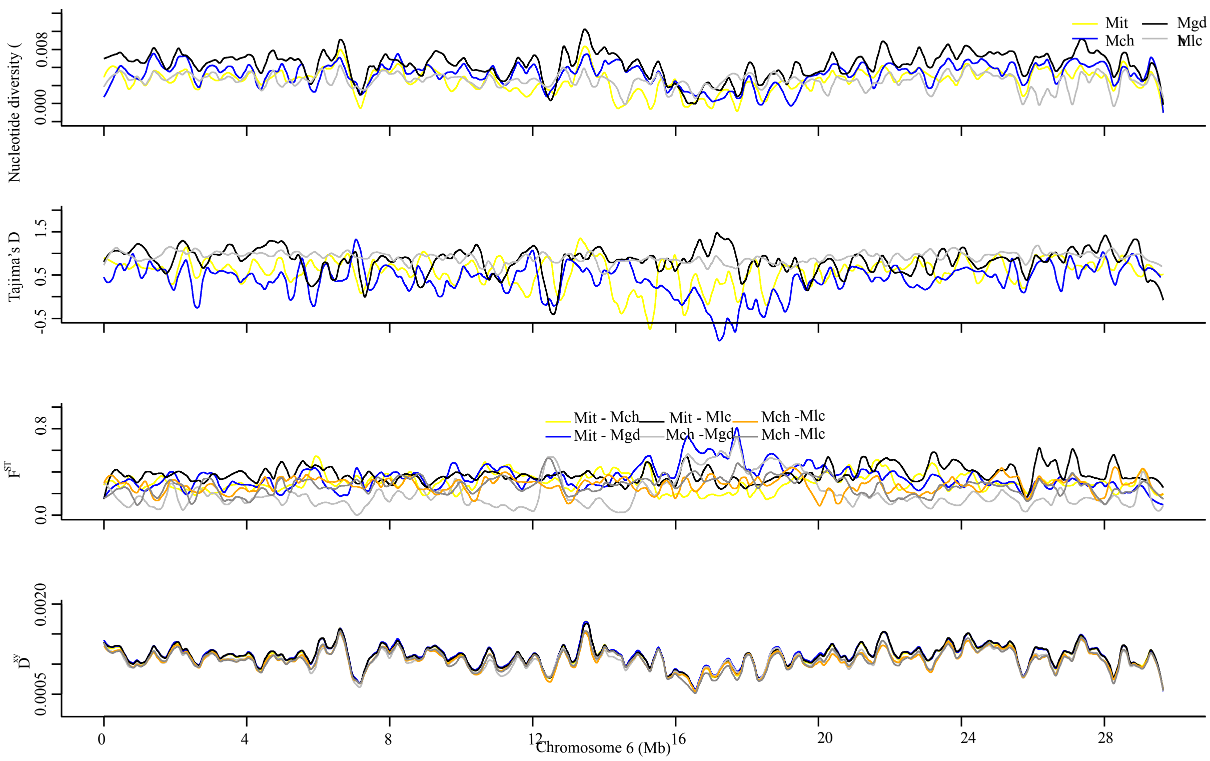

Nucleotide diversity (
0.000
0.008
Mit
Mgd
Mch
Mlc
Tajima's D
-0.5
0.5
1.5
Mit - Mch
Mit - Mlc
Mch -Mlc
Mit - Mgd
Mch -Mgd
Mch -Mlc
F
ST
0.0
0.8
D
xy
0.0005
0.0020
0
4
8
12
16
20
24
28
Chromosome 6 (Mb)

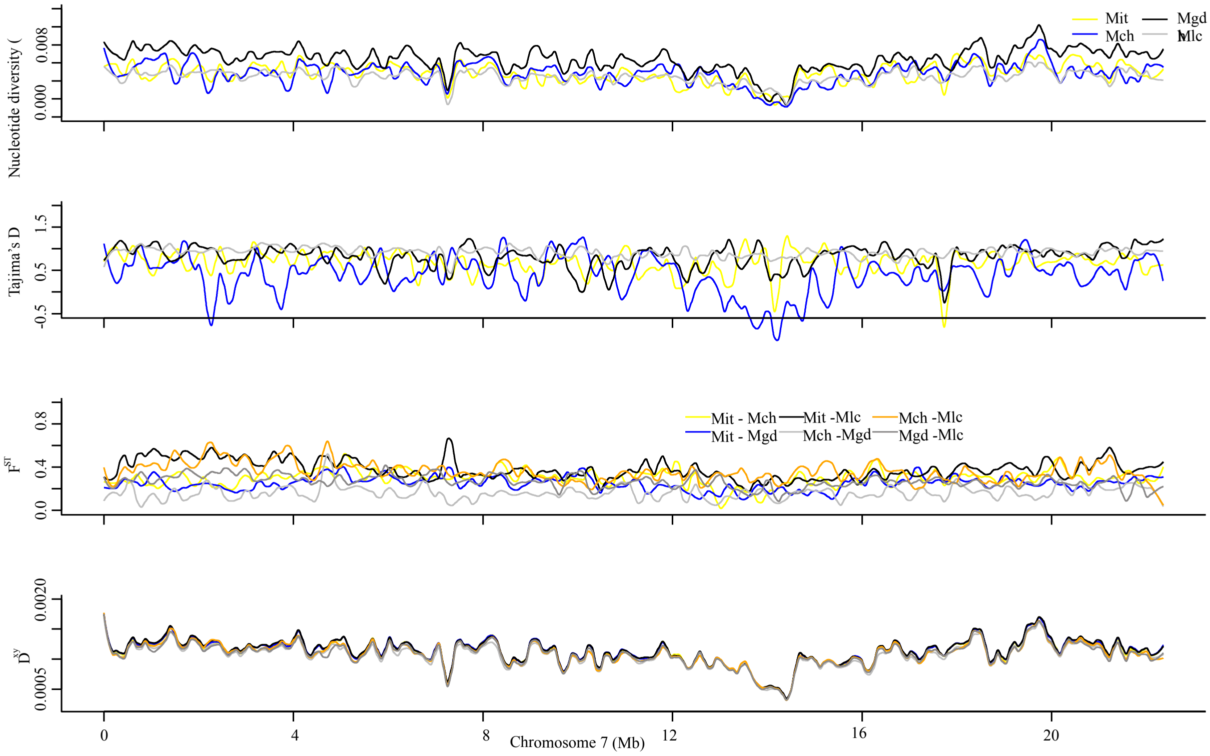

Nucleotide diversity (
0.000
0.008
Mit
Mgd
Mch
Mlc
Tajima's D
-0.5
0.5
1.5
Mit - Mch
Mit -Mlc
Mch -Mlc
Mit - Mgd
Mch -Mgd
Mgd -Mlc
F
ST
0.0
0.4
0.8
D
xy
0.0005
0.0020
0
4
8
12
16
20
Chromosome 7 (Mb)

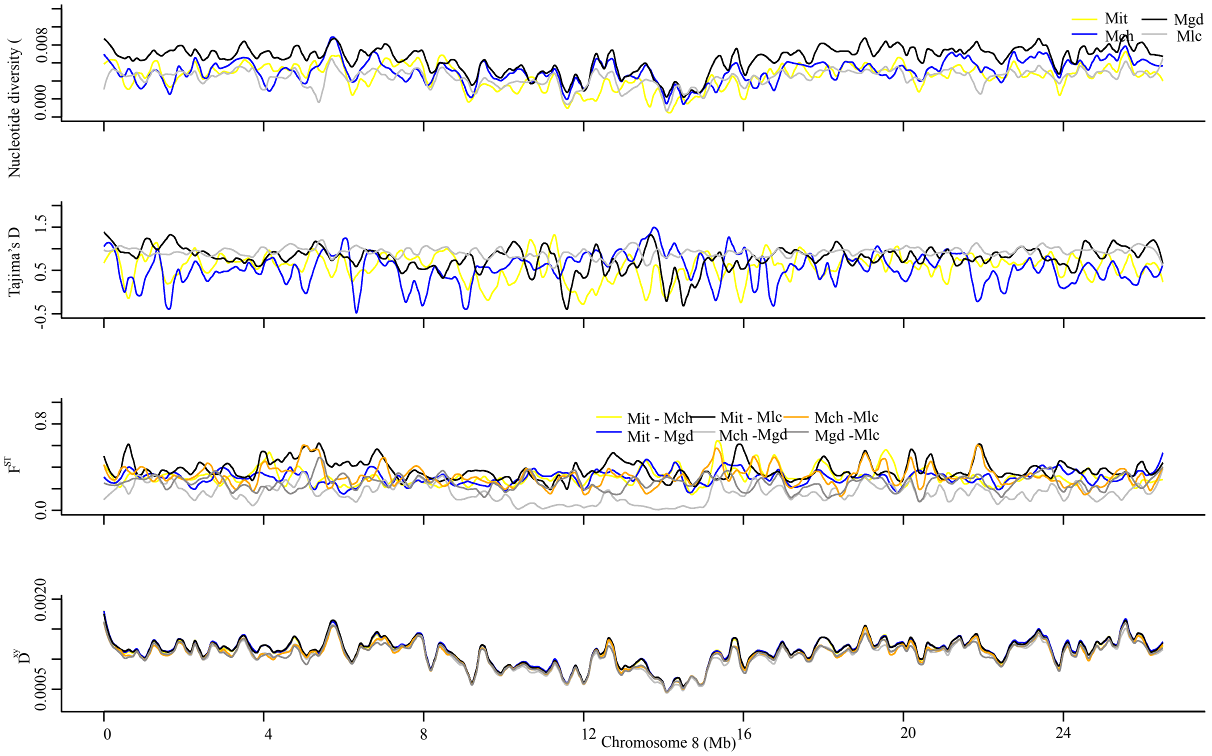
Nucleotide diversity (
0.000
0.008
Mit
Mch
Mgd
Mlc
Tajima's D
-0.5
0.5
1.5
Mit - Mch
Mit - Mlc
Mch -Mlc
Mit - Mgd
Mch -Mgd
Mgd -Mlc
F ST
0.0
0.8
D xy
0.0005
0.0020
0
4
8
12
16
20
24
Chromosome 8 (Mb)

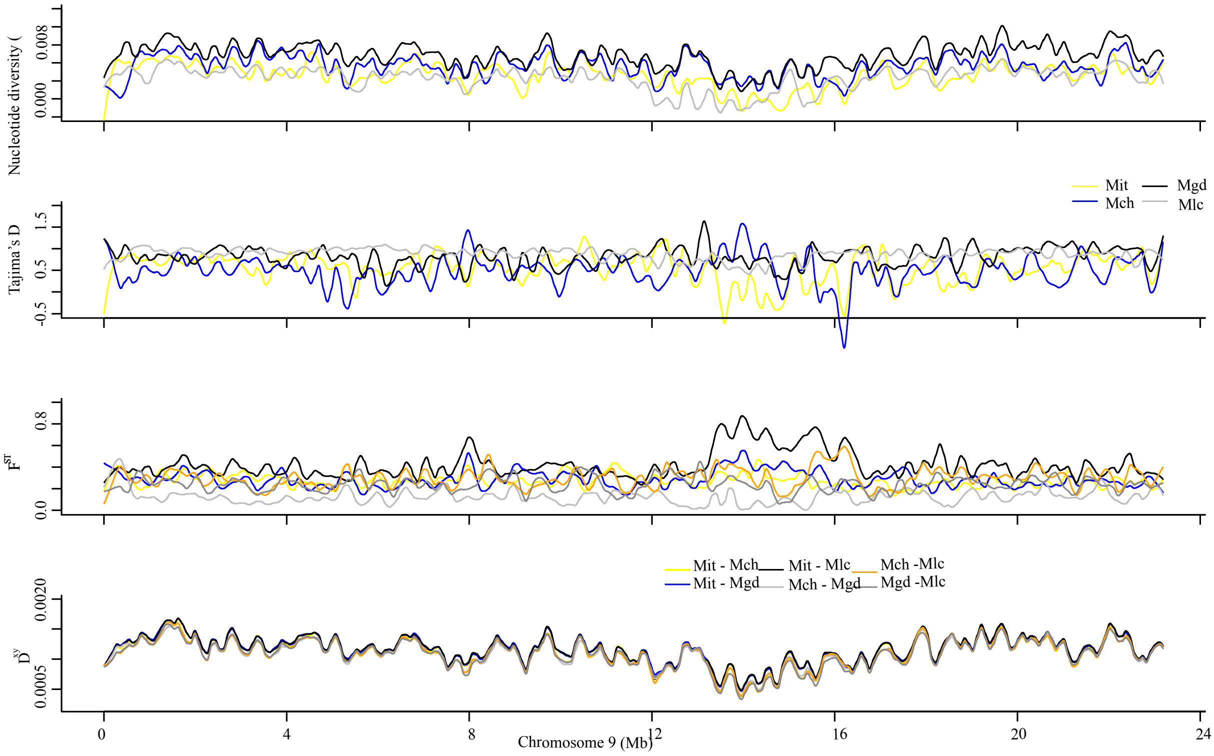
Nucleotide diversity (
0.000
0.008
Mit
Mch
Mgd
Mlc
Tajima's D
-0.5
0.5
1.5
F ST
0.0
0.8
Mit - Mch
Mit - Mlc
Mch -Mlc
Mit - Mgd
Mch - Mgd
Mgd -Mlc
D xy
0.0005
0.0020
0
4
8
12
16
20
24
Chromosome 9 (Mb)

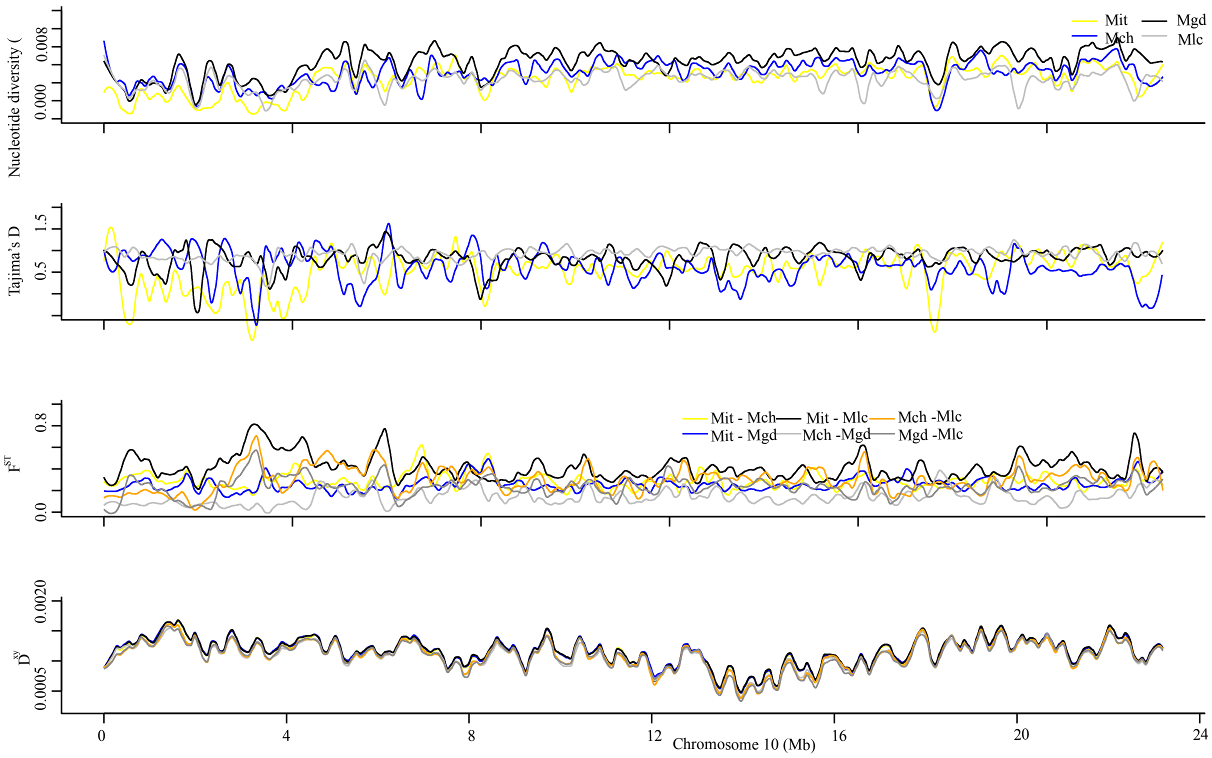
Nucleotide diversity (
0.000
0.008
Mit
Mch
Mgd
Mlc
Tajima's D
0.5
1.5
FST
0.0
0.8
Mit - Mch
Mit - Mlc
Mch -Mlc
Mit - Mgd
Mch -Mgd
Mgd -Mlc
Dxy
0.0005
0.0020
0
4
8
12
16
20
24
Chromosome 10 (Mb)

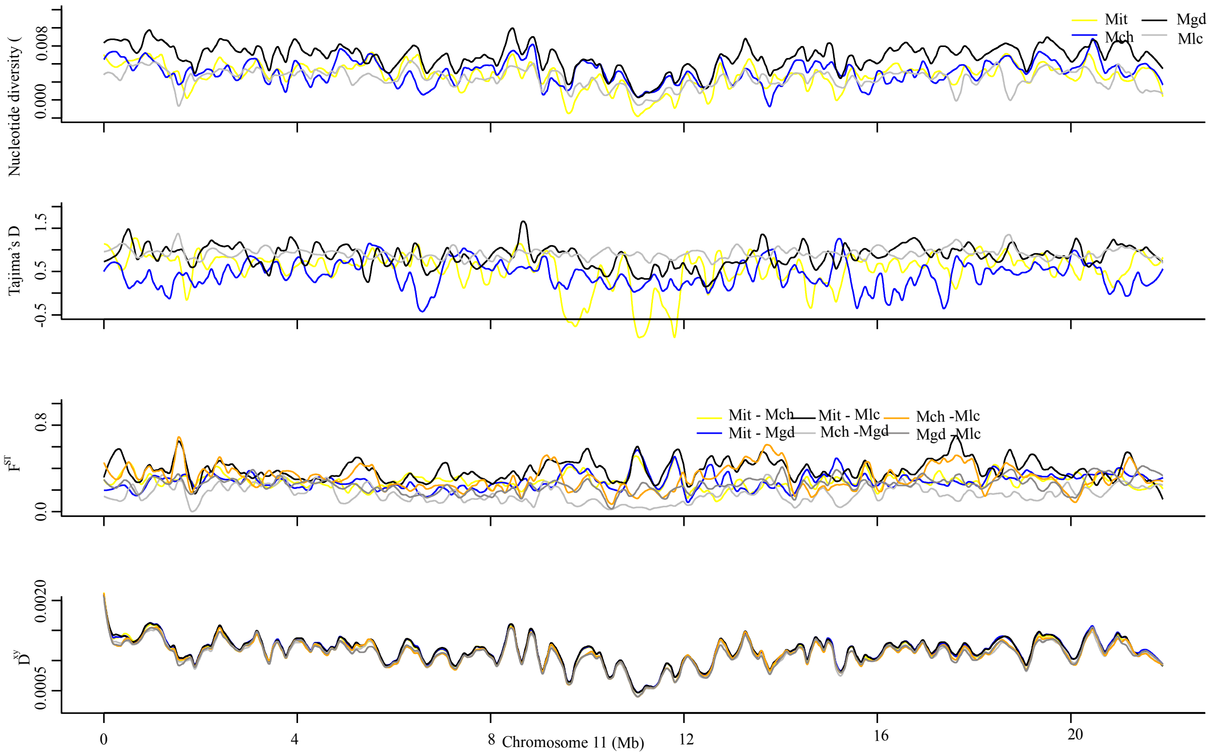
Nucleotide diversity (
0.000
0.008
Mit
Mch
Mgd
Mlc
Tajima's D
-0.5
0.5
1.5
FST
0.0
0.8
Mit - Mch
Mit - Mlc
Mch -Mlc
Mit - Mgd
Mch -Mgd
Mgd -Mlc
Dxy
0.0005
0.0020
0
4
8
12
16
20
Chromosome 11 (Mb)

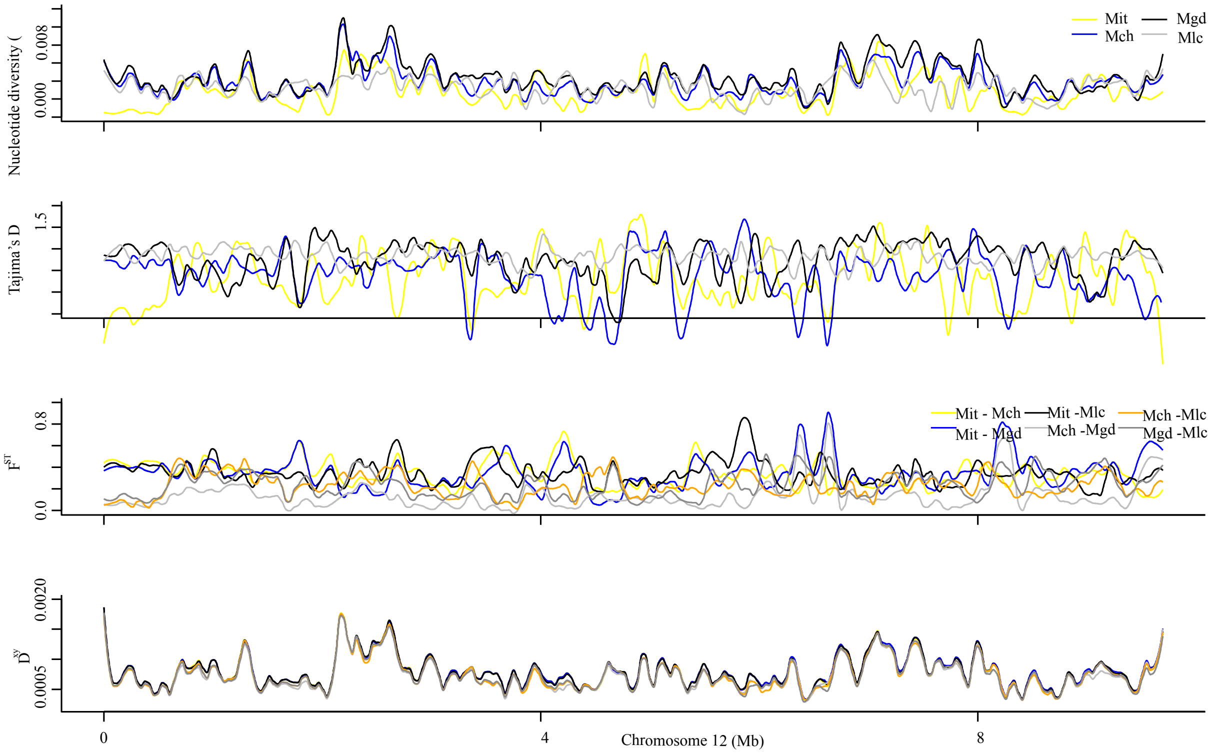

Figure S2 The distribution of average pairwise nucleotide diversity θ_π, and Tajima's D, Wright's fixation index F_{ST} and absolute genetic divergence D_{xy} across the chromosome 2 ~12 with 20k b overlapped window size and 2kb step size among four *Musa itinerans* varieties; The the varieties were designated as: *Mit*: *Musa itinerans* var *itinerans*; *Mch*: *Musa itinerans* var *guangdongensis*; *Mgd*: *Musa itinerans* var *chinensis*; *Mlc*: *Musa itinerans* var *lechangensi.*
